# Medical Student Attitudes toward Complementary, Alternative and Integrative Medicine

**DOI:** 10.1093/ecam/nep195

**Published:** 2011-04-14

**Authors:** Ryan B. Abbott, Ka-Kit Hui, Ron D. Hays, Jess Mandel, Michael Goldstein, Babbi Winegarden, Dale Glaser, Laurence Brunton

**Affiliations:** ^1^Center for East-West Medicine, Department of Medicine, David Geffen School of Medicine, University of California, Los Angeles, CA, USA; ^2^School of Medicine, University of California, San Diego, CA, USA; ^3^Yale Law School, New Haven, CT, USA; ^4^Division of General Internal Medicine and Health Services Research, David Geffen School of Medicine, University of California, Los Angeles, CA, USA; ^5^RAND Corporation, Santa Monica, CA, USA; ^6^Department of Medicine, University of California, San Diego, CA, USA; ^7^Department of Community Health Sciences, School of Public Health, University of California, Los Angeles, CA, USA; ^8^Division of Medical Education, University of California, San Diego, CA, USA; ^9^Department of Psychology, San Diego State University, CA, USA; ^10^Department of Pharmacology, University of California, San Diego, CA, USA

## Abstract

While the use of complementary, alternative and integrative medicine (CAIM) is substantial, it continues to exist at the periphery of allopathic medicine. Understanding the attitudes of medical students toward CAIM will be useful in understanding future integration of CAIM and allopathic medicine. This study was conducted to develop and evaluate an instrument and assess medical students' attitudes toward CAIM. The Complementary, Alternative and Integrative Medicine Attitudes Questionnaire (CAIMAQ) was developed by a panel of experts in CAIM, allopathic medicine, medical education and survey development. A total of 1770 CAIMAQ surveys (51% of US medical schools participated) were obtained in a national sample of medical students in 2007. Factor analysis of the CAIMAQ revealed five distinct attitudinal domains: desirability of CAIM therapies, progressive patient/physician health care roles, mind-body-spirit connection, principles of allostasis and a holistic understanding of disease. The students held the most positive attitude for the “mind-body-spirit connection” and the least positive for the “desirability of CAIM therapies”. This study provided initial support for the reliability of the CAIMAQ. The survey results indicated that in general students responded more positively to the principles of CAIM than to CAIM treatment. A higher quality of CAIM-related medical education and expanded research into CAIM therapies would facilitate appropriate integration of CAIM into medical curricula. The most significant limitation of this study is a low response rate, and further work is required to assess more representative populations in order to determine whether the relationships found in this study are generalizable.

## 1. Introduction

### 1.1. Complementary and Alternative Medicine

The term complementary and alternative medicine (CAM) describes a group of health care systems, practices and products not presently considered to be part of allopathic medicine [1]. While systems of CAM (such as chiropractic, ayurveda, homeopathy and naturopathy) display considerable diversity, they share many of the same core values [2]. CAM systems are characterized by a holistic and highly individualized approach to patient care, an emphasis on maximizing the body's inherent healing ability, involving patients as active participants in their own care, addressing physical, mental and spiritual attributes of a disease, and placing a strong emphasis on preventative medicine [2–7].

### 1.2. The Use of CAM in the USA Is Substantial

A recent study of CAM use in the general population reported that in 2007 almost 4 out of 10 adults had used some form of CAM within the past year [1]. In 1998, it was estimated the US public spent between $36 and $47 billion on CAM therapies, with ∼$12–$20 billion of that total spent out-of-pocket for professional CAM services. (This was more than the out-of-pocket fees for all hospitalizations in that year, and about half the amount paid for all out-of-pocket physician services) [3].

### 1.3. Integrative Medicine

Integrative medicine incorporates aspects of both CAM and allopathic medicine; generally it combines allopathic medical therapies with those of CAM that have high-quality scientific evidence of safety and effectiveness [1]. However, like CAM, the practice of integrative medicine is diverse. Specific techniques of CAM can be integrated into a disease-centered model; for example, an orthopedic surgeon may use acupuncture locally to reduce inflammation. This model of integration has resulted in some CAM techniques becoming mainstream, such as patient support groups [1].

Beyond a piecemeal absorption of CAM modalities into an allopathic model of medicine, integrative medicine may provide a new paradigm that incorporates core CAM values [2]. For example, integrative medicine may be characterized by a humanistic, relationship-centered partnership approach to care, a focus on biological, psychological, social and spiritual influences to pathology, and an emphasis on providing hope, education and therapeutic approaches that match an individual's worldview [2].

### 1.4. Medical Student Attitudes

The extent to which integration between CAM and allopathic medicine will occur in the future, as well as the nature of this integration, will be greatly influenced by the attitudes of physicians [8]. However, most physicians know little about CAM [9, 10]. Investigating medical student attitudes toward CAM is, therefore, important in assessing the possibility that this may change. Student attitudes and beliefs may have a strong impact on the way medical students ultimately practice medicine [11]. In previous studies, medical students have consistently expressed interest in gaining more exposure to CAM [12, 13], and medical schools are becoming aware of the need to provide CAM-related education [9]. Most US medical schools now offer coursework addressing CAM [14]. Investigating medical student attitudes toward CAM is also important because in view of the high use of CAM integrative medicine may become a major feature of mainstream health care [8]. This study measures attitudes not only toward CAM, but also toward integrative medicine. CAIM directly involves medical doctors in bridging the gap between allopathic medicine and CAM.

### 1.5. Prior Studies of Medical Student Attitudes toward CAIM

A comprehensive literature review was performed in order to identify prior surveys investigating attitudes, and specifically medical student attitudes, toward CAIM. These earlier studies were generally performed at single sites using self-developed instruments and did not report information about the reliability and validity of the measures [6, 13, 15–27].

The 29-item Integrative Medicine Attitudes Questionnaire (IMAQ), developed at the University of Arizona, was the first such instrument evaluated for psychometric properties [28]. Analyses of the 196 completed IMAQ surveys revealed two underlying domains: (i) openness to new ideas and paradigms and (ii) value of both introspection and relationship to patient. A second study using the same instrument with 272 US medical students yielded an internal consistency reliability of 0.83 for a scale formed from all the IMAQ items [6]. These studies were later criticized for assuming, by using orthogonal rotation, that the factors were not correlated with each other. In addition, the two factors only explained 38% of the variance in responses [29]. Portions of the IMAQ were also used in a survey of medical students at UCLA [30].

A modified version of the IMAQ was developed for a six-school, international study of 604 first-year medical students [29]. A confirmatory factor analysis revealed three distinct scales: (i) attitudes toward holism, (ii) attitudes toward the effectiveness of CAM and (iii) attitudes toward introspection and doctor-patient relationship. However, the internal consistency reliability coefficients (0.41–0.71) and the fit indices (e.g., CFI = 0.887, SRMR = 0.0632) for the three-factor model were unimpressive.

### 1.6. Study Objectives

The purpose of this research was to develop and evaluate a new instrument to assess attitudes toward CAM among US medical students.

## 2. Methods

### 2.1. Study Approval

The protocol and informed consent forms were reviewed and approved by the Institutional Review Board (IRB approval #060557) at the University of California at San Diego (UCSD) for 2006–08.

### 2.2. Instrument Development

To develop the Complementary, Alternative, and Integrative Medicine Attitude Questionnaire (CAIMAQ) (the appendix), a group was formed consisting of academic clinicians, researchers and students from schools of medicine, public health and traditional oriental medicine having expertise in CAIM, allopathic medicine, medical education and survey development.

Based on a review of the literature, as well as personal, clinical and professional expertise, the core CAIM values chosen to be evaluated were:

- A holistic and individualized approach to patient care.
- Belief in the innate healing ability of the body.
- End-of-life care as an opportunity for healing.
- Belief in a spiritual aspect of illness.
- Importance of involving patients as active participants in treatment.
- Provision of hope to patients.
- Humanistic, relationship centered patient-physician interactions.
- Significance of physician well-being and modeling of healthy lifestyles for patients.
- Synergy between CAM and allopathic medicine.
- Sources of evidence other than biomedical research.
- Importance of basic knowledge for physicians regarding CAM therapies.
- Strong emphasis on preventive medicine.
- Focus on health and well-being as distinct from the absence of disease.
- Generally low-invasive nature of treatment.

Items were based on those in the IMAQ, as well as others generated *de novo* or adapted from other instruments, to better represent the core values listed above. Despite the fact that both positive and negative items are frequently used in survey instruments, it is known that combining these items suppresses score reliability [31, 32]. Thus, all the items in this study were positively worded.

The CAM categories used by the National Center for Complementary and Alternative Medicine (NCCAM) were used to measure attitudes toward specific CAM modalities. Previous instruments used these categories to evaluate attitudes toward specific CAM modalities [33, 34]. NCCAM classifies CAM therapies into five categories: whole medical systems (e.g., homeopathic medicine, naturopathic medicine, traditional Chinese medicine, ayurveda), mind-body medicine (e.g., meditation, prayer, art, music, dance), biologically based practices (e.g., dietary supplements, herbal products), manipulative and body-based practices (e.g., chiropractic manipulation, massage) and energy medicine (including both biofield therapies (e.g., Tai Chi, Qi Gong, Reiki, Therapeutic Touch) and bioelectromagnetic-based therapies (e.g., the medical use of pulsed fields, magnetic fields, alternating-current, direct-current fields)) [1]. A glossary of terms adapted from the NCCAM web site was included with the survey to aid participants who were unfamiliar with the terms used.

The CAIMAQ included 30 items. Each item was administered using a seven-point Likert scale, ranging from one (strongly disagree) to seven (strongly agree). A “don't know" option was also explicitly provided and treated as missing data. Following the 30-item CAIMAQ, demographic information was collected on gender, age, race/ethnicity (according to the format recommended by the National Institutes of Health (NIH) [35]), year in medical school, medical school attended, CAM-related medical education and prior exposure to CAM. At the end of the survey, participants were given the opportunity to provide open-ended comments. An online web version of the CAIMAQ was created for this study using SurveyMonkey.

### 2.3. Pilot Study

The CAIMAQ was administered to a group of 56 second-year medical students at the UCSD School of Medicine. Comments were elicited from participants and the data from this administration were analyzed to determine if revisions to the protocol or items were needed before proceeding with national distribution. Some items were revised given the feedback from the pilot study sample that clarified item wording, amended terms in the glossary, eliminated items from the demographic information section and reduced response burden. In addition, the pilot study results were used to hypothesize a potential solution for subsequent confirmatory factor analyses.

### 2.4. National Field Test

A letter introducing the survey was sent electronically to every medical school in the USA in 2007. Specifically, the letter was sent to the Dean of Student Affairs (or equivalent position), the American Medical Student Association (AMSA) officer and the Association of American Medical Colleges Organization of Student Representatives (AAMC OSR) officer at each school. These recipients were then asked to forward an attached electronic recruitment letter to active medical students at their institutions. The electronic recruitment letter contained a link to access the CAIMAQ online. As an incentive for participation, six MP3 players were given at random to participants who completed the survey.

Prior to taking the survey, participants were provided with an informed consent and subject's bill of rights. For a survey to be accepted, participants were required to answer all 30 items of the CAIMAQ and the question “Are you currently a medical student in the USA?" Only the first survey per web address was allowed.

## 3. Results

### 3.1. Response Rate

A total of 1784 completed surveys were obtained from the total population of 68 001 US medical students [36]. Responses were excluded if participants failed to affirmatively answer that they were current US medical students (eight excluded) or if they answered that they attended either a school of osteopathy or a foreign medical school (six excluded). Thus, a total of 1770 responses were included in the final analysis (3% of the US medical student population). Respondents participated from 64 of the 126 medical schools solicited (51%), and participation at each school ranged from 1 to 164. The survey was distributed and the first response collected on April 12, 2007. The survey was closed to further responses and the final response collected on July 3, 2007.

Because of the manner in which the paper was distributed, it is impossible to know the exact response rate. While 51% of medical schools forwarded the electronic recruitment letter to medical students at their institutions, distribution was heterogeneous. Some schools forwarded the electronic recruitment letter individually to students as requested, while other schools posted the recruitment letter on a medical student forum, or in some cases only distributed the survey to a portion of the student body. Consistent information regarding how many students received the recruitment letter was not reported.

### 3.2. Study Population: Demographic and Comparison

Compared to the entire population of US medical students, the CAIMAQ survey population had more females (57 versus 50%) and was more likely to self-identify as white (75 versus 69%) [36, 37]. The reported average age of students who participated in the CAIMAQ survey was 26 years; the national average age of students entering medical school is 24 years (84% of students report graduation in 4 years, 10% in 5 years [38]) (Table 1). Because the CAIMAQ population consisted of students in all years of medical school, the reported age is consistent with the expectation that respondents completing the CAIMAQ would be ~ 2 years older than matriculate age on average. Of the respondents who elected to report their year in school (*n* = 1728), 521 were first-year medical students (29%), 483 were second-year medical students (27%), 409 were third-year medical students (23%) and 315 were fourth-year medical students (18%). 

### 3.3. Medical Student Attitudes toward CAIM

CAIMAQ respondents expressed a wide range of attitudes toward CAIM, from skepticism to enthusiasm (Table 2). On the whole, however, respondents endorsed the importance of CAIM. Seventy-seven percent of participants agreed to some extent that patients whose doctors know about CAM, in addition to conventional medicine, benefit more than those whose doctors are only familiar with conventional medicine. Seventy-four percent of participants agreed to some extent that a system of medicine that integrates therapies of both conventional medicine and CAM would be more effective than either conventional medicine or CAM provided independently. Eighty-four percent agreed to some extent that CAM contains beliefs, ideas and therapies from which conventional medicine could benefit. 

In addition, participants generally expressed positive attitudes toward many of the core principles queried by the CAIMAQ items. Nearly every participant, 99%, agreed to some extent that a patient's mental state influences his or her physical health, and 98% agreed that a patient's treatment should take into consideration all aspects of his or her physical, mental and spiritual health. Eighty-six percent of participants agreed to some extent that doctors who lead balanced lifestyles generate improved patient satisfaction, and 81% agreed that doctors who model a healthy lifestyle generate improved patient outcomes. Eighty percent of respondents agreed to some extent that the focus of a primary care physician should be on promoting health rather than treating disease.

### 3.4. Confirmatory Factor Analysis

Confirmatory factor analysis (CFA) was performed in order to evaluate how items grouped together empirically. We hypothesized *a priori* a three-factor model and an alternative four-factor model based on the results of the IMAQ studies and the pilot study. Data were initially screened in SPSS v. 15.0.1 and then transferred as a tab-delimited file to PRELIS v. 2.54, the preprocessor for LISREL v.8.54, to be used for the CFA [39] and SIMPLIS [40].

The total sample size for this study was *n* = 1770. However, the response “don't know" was treated as missing data; had participant responses with missing data been excluded, it would have resulted in a 40% loss of cases and produced an analyzable sample size of *n* = 1062. The missing data across items ranged from 1 (Item 1: “A patient's treatment should take into consideration all aspects of his or her physical, mental, and spiritual health") to 355 (Item 28: “Therapeutic Touch is credible as a form of treatment"). As the response “don't know" is a legitimate response that does not indicate a positive, negative or neutral attitude to an item, the most reasonable methodological approach was to fill these on the basis of existing data using multiple imputation (MI) [41].

The CFA indicated a slightly better fit for the four-factor structure, though less than adequate fit was evidenced by the global (e.g., SRMR), exact (significant chi-square) and local (e.g., standardized residuals) fit statistics. Information on model specification and identification, results from preliminary analysis, parameter estimation, assessment of model fit, and full interpretation of the results may be accessed online at www.cewm.med.ucla.edu/research/CAIMAQ_data.html.

### 3.5. Exploratory Factor Analysis

**Figure 1:**
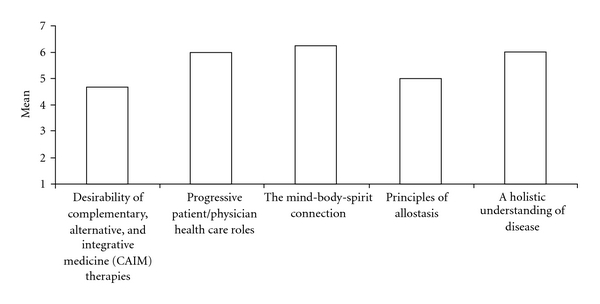
Mean respondent attitudes toward CAIMAQ subscales derived from exploratory factor analysis.

Given the less-than-optimal fit of the hypothesized models, we performed exploratory factor analysis (EFA) using principal components extraction and Promax rotation. The rotated pattern matrix (using the Kaiser Criterion *λ*>1.0 cutoff for factor retention) revealed a five-component solution that explained 51% of the variance and resulted in nine communalities (*h* ^2^) < 0.45. These five factors accounted for the majority of variance; a sixth factor was not substantially meaningful. Promax rotation was used because it utilizes an orthogonal and then oblique rotation [41]. A very similar factorial solution emerged regardless of the type of rotation (orthogonal versus oblique) or extraction (principal components versus principal axis factoring), as well as when varying the power of *k* from two to six. The Promax rotation (*k* = 4) revealed five scales (eigenvalues and percent of variance in parenthesis) that appear to represent attitudes toward (i) the desirability of CAIM therapies (9.693, 32%), (ii) progressive patient/physician health care roles (2.284, 8%), (iii) the mind-body-spirit connection (1.292, 4%), (iv) the principles of allostasis (1.157, 4%) and (v) a holistic understanding of disease (1.041, 3%) (Figure 1). Additional information regarding the EFA is available online at www.cewm.med.ucla.edu/research/CAIMAQ_data.html. 

### 3.6. CAIMAQ Scales

Scale scores were created by averaging responses to the items in each scale. Figure 1 shows that the most positive attitude (mean ± SD) was obtained for “attitudes toward the mind-body-spirit connection" (6.11 ± 0.76) and the lowest for “attitudes toward the desirability of CAIM therapies" (4.69 ± 1.01). Participant attitudes were significantly more positive for factors that queried foundational values of CAIM, such as the mind-body-spirit connection, a holistic understanding of disease and progressive patient/physician health care roles than for the desirability of CAIM therapies. These data seem to support the idea that students are comfortable with the principles of CAIM but may be hesitant to actually endorse providing CAIM.

The relative lack of support for CAIM therapies may be due to, in the words of one participant, a “lack of evidence base for CAM". Comments of this nature were the most frequent in the survey. As another participant stated, “I feel that the biggest…division between alternative medicine and allopathic medicine is…evidence". While 79% of the respondents agreed to some extent that “therapies lacking rigorous support from biomedical research may nevertheless be of value to doctors", the most frequent reason given by physicians for not accepting the use of CAIM is that CAIM therapies are perceived as lacking rigorous scientific support [42, 43].

### 3.7. Year Level for CAIMAQ Subscales

In order to determine whether participant year in medical school was related to CAIMAQ responses, a one-way analysis of variance (ANOVA) was conducted for each of the CAIMAQ subscales to compare them with participant year in medical school. The analysis did not reveal a significant relationship between respondent year in school (MS-I to MS-IV) and any of the CAIMAQ subscales.

### 3.8. Adequacy of CAM-Related Education

Following the CAIMAQ items, participants were asked to respond to a number of questions regarding demographic information, CAM-related medical education and personal CAM use, including the query, “Do you feel that the education you have received regarding complementary and alternative medicine as part of your medical education has been adequate?" Of the respondents who answered this question (*n* = 1720), 39% reported that their CAM-related medical education was adequate.

**Figure 2:**
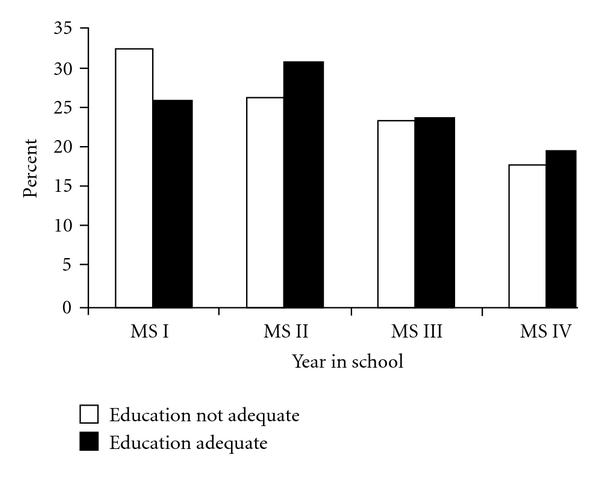
Respondent year in school by perceived adequacy of CAM-related medical education.

In order to determine whether participant year in school was related to the reported adequacy of CAM-related medical education, a chi-square analysis was conducted. The results of the analysis suggest that students are more likely to report their CAM-related education adequate further along in their medical education (*χ* ^2^(3) = 9.41, *P* =  .024, Cramer's *V* = 0.074). This relationship is illustrated in Figure 2. Of note is the relatively high rate of “no" responses for first-year medical students compared to those in other years. 

### 3.9. Personal Use of CAM

A significant percentage of survey respondents reported personal experience with CAM. Forty-nine percent answered that they had treated themselves with CAM, 38% reported having received treatment from a CAM provider (an acupuncturist, chiropractor, etc.) and 14% reported having treated someone else with CAM. The 10 most commonly used CAM therapies during the past year were massage (35%), deep breathing exercises (32%), prayer for health reasons (29%), yoga (28%), meditation (25%), diet-based therapies (20%), herbal medicine (18%), progressive relaxation (15%), aromatherapy (13%) and non-vitamin, non-mineral, natural products (10%). Comments suggest that personal use of CAM was a factor in respondent attitudes, consistent with other research in this area [44]. Some participants also stated that they had a different standard for personal CAM use than for recommending CAM to patients.

## 4. Conclusion

### 4.1. Study Limitations

The most significant limitation of this study is a low response rate, and further work is required to assess more representative populations in order to determine if the relationships found in this study are generalizable. Although the response rate to the CAIMAQ is within the range of response rates for internet surveys [45], the study participants may not be representative of the underlying population. It is possible that the students in the CAIMAQ survey population had more positive, negative or extreme attitudes toward CAIM than students who chose not to participate. Care needs to be exercised in the interpretation of these preliminary results.

The similarity between the demographic information of participants and the general population of medical students is somewhat reassuring. In 51% of medical schools that forwarded the survey, distribution was heterogeneous, and the entire medical student body was unlikely to receive the distribution email. This probably accounted for the higher participation rate at schools such as UCSD, where the faculty and administration endorsed and repeatedly encouraged students to take the survey. At other institutions the survey received no official support. According to anecdotal participant reports, the lack of response may have been due to the distribution method, rather than existing attitudes to the subject matter. In the future, more focused distribution to schools that agree to proactively administer the survey to students should be performed to improve response rate. In addition, greater compensation for participation would likely improve the response rate.

Some of the core CAIM values and CAIMAQ subscales embody principles that are acknowledged by many conventional medical practitioners and educators as crucial to the practice of effective allopathic care. For example, it has been argued that the whole-person approach is simply good medicine, and that a holistic approach to patient care is not the sole domain of CAIM [46].

It is true that a purely allopathic provider may practice a patient-centered, holistic model of health care without the use of any CAIM therapies. However, while conventional medical practitioners may agree in theory with the necessity for such an approach to patient care, in practice it tends to be marginalized [47]. Research suggests that psychosocial factors continue to be overlooked by allopathic practitioners in clinical encounters and underemphasized in medical education [9]. Although the issue continues to be debated, the degree of importance placed on a particular value by CAIM or allopathic medicine may be more significant than whether a value of patient care is strictly CAIM or allopathic.

### 4.2. Adequacy of CAM-Related Education

Compared to the graduating medical students (*n*= 9453) surveyed in the AAMC All Schools Report [38], a relatively larger percentage of CAIMAQ survey participants stated that the time devoted to CAM in medical school was inadequate (61% versus 34%). This difference may be explained in part by variations in question phrasing (e.g., the AAMC item had three responses: inadequate, appropriate and excessive), population bias and/or differences in study populations. The AAMC report was completed only by graduating medical students, whereas the CAIMAQ population comprised students in all years of medical school. Comparing only fourth-year medical students who completed the CAIMAQ narrows the difference somewhat (58% versus 34%). As discussed earlier, fourth-year medical students are significantly more likely to suggest that they received adequate education in CAM than students in earlier years.

### 4.3. Medical School Effects on Attitudes

In open-ended responses, participants reported that their experiences in medical school affected their attitudes toward CAIM. In addition, respondent year in medical school affected the perceived adequacy of CAM-related education; students further along in the educational process were more likely to endorse their CAM-related education to be adequate. Still, >60% of participants were in favor of having more CAIM-related education during their time in medical school. This view accords with the conclusions of a report by the Institute of Medicine, Complementary and Alternative Medicine Use in the United States, which noted that “conventional professionals in particular need enough CAM-related training…so that they can counsel patients in a manner consistent with high-quality comprehensive care" [48, page 8].

However, while the overall quantity of CAIM-related education in US medical schools has steadily increased, the quality of that education has varied significantly [49–53]. Some schools have attempted to incorporate CAIM education by providing elective courses, and as early as in 1998 a survey of 117 of the existing 125 US medical schools found that 64% of the schools offered such courses [52]. Yet only 47% of CAIMAQ survey participants reported that coursework in CAM was offered at their medical school and only 16% of students reported studying CAM as an elective. Almost a quarter of the participants (24%) were uncertain about the availability of CAM-related coursework at their school.

Two themes emerged from participant comments regarding the CAIM education they received in medical school. As one participant stated, “We did take classes in CAM, but they seemed to be of low quality, the lecturers rarely acknowledged the limitations of the therapies." Several respondents commented that the CAIM-related education they received was biased to be either pro- or anti-CAIM, and of relatively poor quality compared to the rest of their medical education. In the words of another participant, “I heard a lot of unscientific nonsense, including from medical students and MDs". Students who perceive their CAIM-related training as inadequate may be significantly less open to addressing CAIM-related issues in their interactions with patients [44]. This suggests the need for a uniform and better informed approach to educating future physicians about CAIM.

Although limited academic research has addressed this area, medical educators have increasingly begun to explore how best to teach CAIM to allopathically trained physicians, as well as to provide guidelines on teaching CAIM to medical students, residents and fellows [54–60].

### 4.4. Concluding Thoughts

Analysis of the CAIMAQ provided evidence of its reliability and construct validity and supports its use in future studies. Further work is required to assess more representative populations in order to determine whether the relationships found in this study are valid. Future trials should also utilize confirmatory factor analysis to further evaluate the structure revealed in this study.

There is a need for emerging physicians to understand and address the practices of CAIM, but the results of this survey suggest that significant obstacles remain. First, while medical students appear receptive to the underlying principles of CAIM, they may perceive CAIM therapies as not being evidence based. From a policy standpoint, this suggests that for CAIM to be appropriately integrated into conventional health care, more research is needed, particularly research that evaluates the mechanisms, safety and cost-effectiveness of CAIM therapies. Although future physicians may be willing to use CAIM themselves, many are unwilling to cross the barrier to recommending or using CAIM in their practice until more assessment has occurred. Lack of studies evaluating the effectiveness of CAIM may be the principal obstacle to integration by mainstream health care practitioners.

Second, medical student education in CAIM must be improved. Development of a more comprehensive and consistent educational approach to teaching medical students about CAIM is necessary if future physicians are to be adequately prepared for their role as health care providers. Just as medical schools have restructured their curricula to reflect the changing practice of medicine and incorporate new fields of study such as HIV/AIDS, gene therapy and immunology, medical educators must recognize the importance of educating future doctors in health care systems outside of conventional medicine. This education must be guided by appropriate evidence, good science and an understanding of the differences inherent in various forms of medicine.

## Figures and Tables

**Table 1:** Demographic characteristics of respondents to the CAIMAQ^a^.

	CAIMAQ population (*n* = 1770)	Total population of US medical students (*n* = 68 343)
*What is your gender?*		
Answer options	Response percent (*n* = 1743)	Response percent [34] (*n* = 68 343)
Male (%)	42.7	49.9
Female (%)	57.3	50.1

*Respondent age*		
Answer options	Response percent (*n* = 1745)	Response percent [35] (*n* = 16 875)
Mean	25.8 years	Mean age at matriculation: 23.7
Standard deviation	3.62	

*What is your ethnicity?*		
Answer options	Response percent (*n* = 1718)	Response percent [34] (*n* = 68 343)
Hispanic or Latino (%)	5.59	7.01
Not Hispanic or Latino (%)	94.4	91.6
Undetermined (%)		1.39

*What is your race? (You many select multiple answers for this question)*		
Answer options	Response percent (*n* = 1714)	Response percent [34] (*n* = 68 343)
White (%)	74.5	68.9
Black or African American	3.4	8.02
Asian (%)	16.9	22.7
Native Hawaiian or other Pacific Islander (%)	0.2	0.3
American Indian or Alaskan Native (%)	0.6	1.2
Multiple races (%)	4.4	N/A

^a^Respondents were US medical students in all years of medical school recruited from 64 of the 126 medical schools solicited. N/A for the total population data.

**Table 2:** Summary of CAIMAQ items and exploratory factor analysis (*n* = 1770 all items).

*Factor 1. Attitudes toward the desirability of CAIM therapies* (*α* = 0.90)	
Item 3: Patients whose doctors know about complementary and alternative medicine, in addition to conventional medicine, benefit more than those whose doctors are only familiar with conventional medicine.	
Item 4: When systems of alternative medicine (such as traditional Chinese medicine) are found to be efficacious in treatment of a disease, doctors should recommend them even though these systems may rely on unknown mechanisms.	
Item 6: Therapies lacking rigorous support from biomedical research (randomized controlled trials, etc.) may nevertheless be of value to doctors.	
Item 8: A system of medicine that integrates therapies of both conventional medicine and complementary and alternative medicine would be more effective than either conventional medicine or complementary and alternative medicine provided independently.	
Item 10: The use of herbal products represents a legitimate form of medicine that can treat a wide variety of disease.	
Item 15: Complementary and alternative medicine contains beliefs, ideas and therapies from which conventional medicine could benefit.	
Item 16: Chiropractic care can be a valuable method for resolving a wide variety of musculoskeletal problems.	
Item 18: Massage therapy can lead to objective improvements in long-term outcomes for patients.	
Item 25: Doctors should consider referring patients to alternative health care providers such as homeopaths or naturopaths for conditions poorly managed by conventional medicine.	
Item 27: It is ethical for doctors to recommend therapies to patients that involve the use of subtle energy fields in and around the body for medical purposes.	
Item 28: Therapeutic Touch is credible as a form of treatment.	
Item 30: Treatments of complementary and alternative medicine tend to be less invasive that those of conventional medicine, and may help to reduce the risk of side effects and iatrogenesis.	

*Factor 2. Attitudes toward progressive patient/physician health care roles* (*α* = 0.79)	
Item 13: Patients who express themselves through creative outlets such as art, music or dance may achieve significant health benefits through these activities.	
Item 14: Doctors who lead a balanced lifestyle (i.e., attending to their own health, social, family and spiritual needs, as well as interests beyond medicine) generate improved patient satisfaction.	
Item 20: A strong relationship between patients and their doctors is a valuable therapeutic intervention that leads to improved outcomes.	
Item 21: Doctors who model a healthy lifestyle (i.e., follow their own advice) generate improved patient outcomes.	
Item 22: Whenever reasonable, a physician should provide patients with hope and a positive attitude toward healing.	
Item 23: A patient who is an active participant in his or her care is likely to experience improved outcomes compared with a patient who is a passive participant.	
Item 24: Nutritional counseling and dietary/food supplements can be effective in the treatment of pathology.	

*Factor 3. Attitudes toward the mind-body-spirit connection* (*α* = 0.70)	
Item 1: A patient's treatment should take into consideration all aspects of his or her physical, mental and spiritual health.	
Item 5: Prayer, for oneself or others, can benefit quality of life and disease outcomes.	
Item 7: The spiritual beliefs of patients play an important role in their recovery.	
Item 11: A patient's mental state influences his or her physical health.	

*Factor 4. Attitudes toward the principles of allostasis* (*α* = 0.50)	
Item 2: The focus of a primary care physician should be on promoting health rather than treating disease.	
Item 12: Disease occurs when the body's innate ability to heal itself becomes compromised.	
Item 19: The innate self-healing capacity of patients often determines the outcome of illness regardless of treatment interventions.	

*Factor 5. Attitudes toward a holistic understanding of disease* (*α* = 0.66)	
Item 9: End-of-life care should be valued as an opportunity for patients to heal.	
Item 17: A patient with a terminal illness can experience mental and spiritual healing in being at peace with himself or herself.	
Item 26: Even in the absence of clinically significant disease, a person can experience a vast range in terms of physical health.	
Item 29: Disease can be viewed as an opportunity for personal change and growth.	

**Table:** (a)

Item	Response options
What is your gender?	- Male - Female

What is your age:	- Numerical response (18–99)

What is your ethnicity?	- Hispanic or Latino - Not Hispanic or Latino

What is your race? (You many select multiple answers for this question)	- White - Black or African American - Asian - Native Hawaiian or Other Pacific Islander - American Indian or Alaskan Native

Are you a medical student in the USA?	- Yes - No

What is your year in medical school?	- MS-I - MS-II - MS-III - MS-IV - NA

What medical school do you attend?	- List response of all US medical schools - Other (with open response)

Is coursework in CAM offered at your medical school?	- Yes - No - Do not know

Would you like to receive more education about CAM as part of your medical education?	- Yes - No

Do you feel that the education you have received regarding CAM as part of your	- Yes
medical education has been adequate?	- No

Have you studied CAM? (You may select more than one answer for this question.)	- As part of the core coursework at your medical school - As an elective at your medical school - Outside of your medical school - Never

Have you ever treated yourself with CAM?	- Yes - No

Have you ever treated someone else with CAM?	- Yes - No

Have you ever received treatment from a provider of CAM(e.g., an acupuncturist, a chiropractor, etc.)	- Yes - No

Have you ever personally used any of the following forms of CAM?	

**Table:** (b)

	Never	More than 12 months ago	Within the past 12 months
Acupuncture			
Aromatherapy			
Ayurveda			
Biofeedback			
Chelation therapy			
Chiropractic care			
Deep breathing exercises			
Diet-based therapies			
Energy healing therapy/Reiki			
Folk medicine			
Guided imagery			
Herbal medicine			
Homeopathic treatment			
Hypnosis			
Massage			
Magnet therapy			
Meditation			
Megavitamin therapy			
Naturopathy			
Nonvitamin, nonmineral, natural products			
Prayer for health reasons			
Progressive relaxation			
Reflexology			
Qi gong			
Tai chi			
Yoga			

Do you have any comments?	Open response		

## Appendices

### 6. CAIMAQ Instrument

For each of the 30 items below, please indicate how strongly you identify with the associated statement by selecting one of the answer choices. For example, selecting “strongly agree" would indicate that you highly believe the statement is accurate; selecting “strongly disagree" would indicate that you highly believe the statement is inaccurate. If you do not feel that you are able to fairly answer an item, a “don't know” option is provided.

If there are any words or therapies you are unfamiliar with, you will find a description in the glossary of terms at the bottom of the page (listed alphabetically).

1. A patient's treatment should take into consideration all aspects of his or her physical, mental and spiritual health.
2. The focus of a primary care physician should be on promoting health rather than treating disease.
3. Patients whose doctors know about complementary and alternative medicine, in addition to conventional medicine, benefit more than those whose doctors are only familiar with conventional medicine.
4. When systems of alternative medicine (such as traditional Chinese medicine) are found to be efficacious in treatment of a disease, doctors should recommend them even though these systems may rely on unknown mechanisms.
5. Prayer, for oneself or others, can benefit quality of life and disease outcomes.
6. Therapies lacking rigorous support from biomedical research (randomized controlled trials, etc.) may nevertheless be of value to doctors.
7. The spiritual beliefs of patients play an important role in their recovery.
8. A system of medicine that integrates therapies of both conventional medicine and complementary and alternative medicine would be more effective than either conventional medicine or complementary and alternative medicine provided independently.
9. End-of-life care should be valued as an opportunity for patients to heal.
10. The use of herbal products represents a legitimate form of medicine that can treat a wide variety of disease.
11. A patient's mental state influences his or her physical health.
12. Disease occurs when the body's innate ability to heal itself becomes compromised.
13. Patients who express themselves through creative outlets such as art, music or dance may achieve significant health benefits through these activities.
14. Doctors who lead a balanced lifestyle (i.e., attending to their own health, social, family and spiritual needs, as well as interests beyond medicine) generate improved patient satisfaction.
15. Complementary and alternative medicine contains beliefs, ideas and therapies from which conventional medicine could benefit.
16. Chiropractic care can be a valuable method for resolving a wide variety of musculoskeletal problems.
17. A patient with a terminal illness can experience mental and spiritual healing in being at peace with himself or herself.
18. Massage therapy can lead to objective improvements in long-term outcomes for patients.
19. The innate self-healing capacity of patients often determines the outcome of illness regardless of treatment interventions.
20. A strong relationship between patients and their doctors is a valuable therapeutic intervention that leads to improved outcomes.
21. Doctors who model a healthy lifestyle (i.e., follow their own advice) generate improved patient outcomes.
22. Whenever reasonable, a physician should provide patients with hope and a positive attitude toward healing.
23. A patient who is an active participant in his or her care is likely to experience improved outcomes compared with a patient who is a passive participant.
24. Nutritional counseling and dietary/food supplements can be effective in the treatment of pathology.
25. Doctors should consider referring patients to alternative health care providers such as homeopaths or naturopaths for conditions poorly managed by conventional medicine.
26. Even in the absence of clinically significant disease, a person can experience a vast range in terms of physical health.
27. It is ethical for doctors to recommend therapies to patients that involve the use of subtle energy fields in and around the body for medical purposes.
28. Therapeutic Touch is credible as a form of treatment.
29. Disease can be viewed as an opportunity for personal change and growth.
30. Treatments of complementary and alternative medicine tend to be less invasive that those of conventional medicine, and may help to reduce the risk of side effects and iatrogenesis.

### 7. Glossary of Terms

Chiropractic—A CAM alternative medical system. It focuses on the relationship between bodily structure (primarily that of the spine) and function, and how that relationship affects the preservation and restoration of health. Chiropractors use manipulative therapy as an integral treatment tool.

#### 7.1. CAM

A group of diverse medical and health care systems, practices and products that are not presently considered to be part of conventional medicine.

#### 7.2. Conventional Medicine

Conventional medicine is medicine as practiced by holders of M.D. (medical doctor) or D.O. (doctor of osteopathy) degrees and by their allied health professionals, such as physical therapists, psychologists and registered nurses. Other terms for conventional medicine include allopathy, or western, mainstream, orthodox and regular medicine, and biomedicine. Some conventional medical practitioners are also practitioners of CAM.

#### 7.3. Dietary Supplements

A dietary supplement is a product (other than tobacco) taken by mouth that contains a “dietary ingredient" intended to supplement diet. Dietary ingredients may include vitamins, minerals, herbs or other botanicals, amino acids and substances such as enzymes, organ tissues and metabolites. Dietary supplements come in many forms, including extracts, concentrates, tablets, capsules, gel caps, liquids and powders.

#### 7.4. Energy Therapies

Energy therapies involve the use of energy fields. They are of two types. Biofield therapies are intended to affect energy fields that may surround and penetrate the human body. Some forms of energy therapy manipulate biofields by applying pressure and/or manipulating the body by placing the hands in, or through, these fields. Examples of this include Qi Gong, Reiki and Therapeutic Touch. Bioelectromagnetic-based therapies involve the unconventional use of electromagnetic fields such as pulsed fields, magnetic fields or alternating- and direct-current fields.

#### 7.5. Herbal Supplements

A type of dietary supplement that contains herbs, either alone or in combination. An herb (also called a botanical) is a plant or plant part used for its scent, flavor and/or therapeutic properties. Herbal supplements (formulas) are used in systems of alternative medicine, such as traditional Chinese medicine and Ayurveda, under the guidance of trained practitioners.

#### 7.6. Homeopathic Medicine

A CAM medical system. In homeopathic medicine, there is a belief that “like cures like", meaning that small, highly diluted quantities of medicinal substances are given to cure symptoms or disease, when the same substances given at higher or more concentrated doses would actually cause those symptoms.

#### 7.7. Iatrogenisis

This refers to the inadvertent and preventable induction of disease or complications as a result of medical treatment or procedures.

#### 7.8. Massage Therapy

Massage therapists manipulate muscle and connective tissue to enhance function of those tissues and promote relaxation and well-being.

#### 7.9. Naturopathic Medicine

Also termed naturopathy, this is a CAM medical system. Naturopathic medicine postulates that there is a healing power in the body that establishes, maintains and restores health. Practitioners work with the patient with the goal of supporting this power through treatments such as nutrition and lifestyle counseling, dietary supplements, medicinal plants, exercise, homeopathy and treatments from traditional Chinese medicine.

#### 7.10. Therapeutic Touch

This is derived from an ancient technique called laying-on of hands. It is based on the premise that it is the healing force of the therapist that affects the patient's recovery; healing is promoted when the body's energies are in balance; and, by passing their hands over the patient, healers can identify energy imbalances.

#### 7.11. Traditional Chinese Medicine (TCM)

TCM is the current name for an ancient system of health care from China. TCM is based on a concept of balanced qi, or vital energy, that is believed to flow throughout the body. Qi is postulated to regulate a person's spiritual, emotional, mental and physical balance and to be influenced by the opposing forces of yin (negative energy) and yang (positive energy). Disease is proposed to result from the flow of qi being disrupted and yin and yang becoming imbalanced. Among the components of TCM are herbal and nutritional therapy, restorative physical exercise, meditation, acupuncture and remedial massage.

### 7.12. Demographic Information

For each of the questions shown in Table 3, please answer as accurately as possible. Your answers will be completely anonymous and in no way connected to you personally.
